# Protein Structure Idealization: How accurately is it possible to model protein structures with dihedral angles?

**DOI:** 10.1186/1748-7188-8-5

**Published:** 2013-02-25

**Authors:** Xuefeng Cui, Shuai Cheng Li, Dongbo Bu, Babak Alipanahi, Ming Li

**Affiliations:** 1 University of Waterloo, Ontario, Canada; 2 City University of Hong Kong, Hong Kong, China; 3 Chinese Academy of Sciences, Beijing, China

**Keywords:** Protein structure idealization, Ideal bond length and angle, Dihedral angle space

## Abstract

Previous studies show that the same type of bond lengths and angles fit Gaussian distributions well with small standard deviations on high resolution protein structure data. The mean values of these Gaussian distributions have been widely used as ideal bond lengths and angles in bioinformatics. However, we are not aware of any research done to evaluate how accurately we can model protein structures with dihedral angles and ideal bond lengths and angles.

Here, we introduce the protein structure idealization problem. We focus on the protein backbone structure idealization. We describe a fast *O*(*nm*/*ε*) dynamic programming algorithm to find an idealized protein backbone structure that is approximately optimal according to our scoring function. The scoring function evaluates not only the free energy, but also the similarity with the target structure. Thus, the idealized protein structures found by our algorithm are guaranteed to be protein-like and close to the target protein structure.

We have implemented our protein structure idealization algorithm and idealized the high resolution protein structures with low sequence identities of the CULLPDB_PC30_RES1.6_R0.25 data set. We demonstrate that idealized backbone structures always exist with small changes and significantly better free energy. We also applied our algorithm to refine protein pseudo-structures determined in NMR experiments.

## Background

When studying the functions of a protein, it is crucial to know the three-dimensional structure consisting of the Cartesian coordinates of all the atoms of the protein. These atoms are bonded together by inter-atomic forces called chemical bonds. It has been observed that the bond lengths and angles of the same type assume a Gaussian distribution with a small standard deviation (STDEV) in high resolution protein structure data. Typically, the bond lengths on protein backbones have STDEVs between 0.019Å and 0.033Å and the bond angles on protein backbones have STDEVs between 1.5° and 2.7° [1,2]. These results suggest the possibility of modeling protein structures with the mean values of bond lengths and angles, which are often referred to as *ideal values*.

Ideal bond lengths and angles have been widely used in nuclear magnetic resonance (NMR) protein structure determination [3,4] and in protein structure prediction [5-9]. Moreover, stereochemical restraints are also used in X-ray protein structure determination [10,11]. In protein structure prediction, the main advantage of using ideal bond lengths and angles is a reduction in the search space for the target protein structure [12,13]. Specifically, if the target protein has *n* amino acids, the number of *n*, *C*_*α*_ and *C* atoms on the backbone is 3*n*, and thus the Cartesian search space for the idealized backbone structure has a degree of freedom of 9*n*[12,13]. However, if all bond lengths and angles have ideal values, the protein backbone structure can be represented by a series of bond torsion angles in the feasible bond torsion angle space. In this case, the degree of freedom is reduced to approximately one tenth of that in the Cartesian space [12,13].

Although ideal bond lengths and angles have been widely used and accepted, we are not aware of any research done to evaluate how accurately it is possible to model protein structures with dihedral angles. This motivates us to solve what we call the *protein structure idealization problem*: Given the coordinates of the target protein structure, find the coordinates of the optimal idealized protein structure. Here, an idealized protein structure is a protein structure with bond lengths and angles that are ideal with respect to a given scoring function; the function depends on the resultant structure’s free energy, as well as its similarity with the target structure. Thus, the idealized protein structure is taken to be a protein-like structure that is close to the target protein structure.

First, we solve the protein structure idealization problem by idealizing the backbone structure and then idealizing the side-chain structure. This approach is widely accepted because previous research suggests that the backbone conformation is archived before the side-chain conformations are archived [14]. In our work, *Ω* dihedral angles are rounded to be either 0° or 180°. Some discussions on the properness of idealizing *Ω* dihedral angles can be found in [15,16].

We introduce a novel dynamic programming algorithm with a run-time complexity of *O*(*n*/*ε*^8^), where *ε* is a small constant, to find the optimal idealized protein backbone structure according to our scoring function. In practice, we observed that it is unnecessary to remember the entire dynamic programming table. Thus, with a filtering technique, the run-time complexity is further reduced to *O*(*nm*/*ε*), where *m* is a constant integer.

In our initial study on the protein structure idealization problem, side-chain structures are determined using an exhaustive search which assumes that side-chain structures of different residues are independent from each other. The scoring function is similar to the one we used for backbone structure idealization. In practice, we observe that it is fast to regenerate idealized structures that are similar to a given idealized structure. We also refine the idealized backbone and side-chain structures according to our scoring functions iteratively.

We use our algorithm to evaluate how accurately it is possible to model protein structures with dihedral angles. We idealize all the X-ray protein structures from PDB [17] which satisfy the high resolution and the low sequence identity constraints downloaded on June 6, 2008 [18,19]. The results show that such idealized structures always exist and that they are very similar to the target structures in terms of the root mean square deviation (RMSD) of *C*_*α*_ or all atoms. Moreover, the idealized backbone structures tend to have dDFIRE free energy scores [20,21], which are significantly better than the target structures. The results support our conclusion that it is possible to model protein structures accurately with dihedral angles on all high resolution protein backbone structures.

One application of the protein structure idealization algorithm is to refine protein pseudo-structures either determined in experiments or predicted by computers. We have demonstrated one such case to improve poor (*Φ*,*Ψ*) dihedral angles of protein structures determined by NMR. The experiment result is also consistent with the previous experiment showing that the idealized structure has a small RMSD and better backbone free energy. We discuss several potential applications for our protein structure idealization algorithm in the conclusion.

## Protein backbone structure idealization

Given the target protein backbone structure, we would like to find the optimal idealized backbone structure. For an idealized protein backbone structure, the coordinates of *O*, *H* and *C*_*β*_ backbone atoms can be calculated from the coordinates of *n*, *C*_*α*_ and *C* backbone atoms. Thus, we specifically describe how to generate coordinates of *n*, *C*_*α*_ and *C* atoms in this section. For simplicity, a structure is always referred to as a protein backbone structure unless strictly specified.

### Idealized backbone structure generation

Given the target structure, we would like to generate idealized structures fulfilling two generation goals. First, the idealized structures should be similar to the target structure. Second, each pair of idealized structures should be at least some distance away to avoid redundant computation. Furthermore, we are interested in generating as many of these idealized structures as possible.

Before describing how we fulfill the generation goals, we describe a simple distance metric to measure the distance between two sets of coordinates representing the target protein. Let *P*_*i*_ be a set of coordinates representing the target protein, and $P_{i}^{j}\in P_{i}$ be the coordinate of the *j*-th atom of the target protein. Thus, there is $P_{i}=\{P_{i}^{1},P_{i}^{2},\mathrm{...},P_{i}^{3n}\}$, where *n* is the number of amino acids of the target protein. For simplicity, let *P*_0_ always represent the target structure, and *P*_*i*_ represent a generated idealized structure for *i*>0. Let $D(P_{i}^{k},P_{j}^{k})$ be the Euclidean distance between $P_{i}^{k}$ and $P_{j}^{k}$. We describe the distance between *P*_*i*_ and *P*_*j*_ as the bottleneck distance:

(1) $$D(P_{i},P_{j})=\underset{k}{\max}D(P_{i}^{k},P_{j}^{k}).$$

Using this distance metric, we fulfill both generation goals by satisfying the following generation constraints:

(2) $$\left\{\begin{array}{ll} D(P_{0},P_{i})\leq r & \forall i>0\\ D(P_{i},P_{j})\geq \varepsilon & \forall i,j>0 \end{array}\right).$$

The first generation constraint assumes that the accuracy of the coordinates of the target structure is reasonably good, and no-worse than *r*. If this constraint is satisfied, the distance between the target coordinate and any generated coordinate representing the same atom is upper bounded by *r*. Thus, it is reasonable for any generated idealized structure *P*_*i*_ to be considered similar to target structure *P*_0_. If the second generation constraint is satisfied, for each pair of generated idealized structures, there exists a pair of coordinates, one from each structure representing the same atom, such that they are at least *ε* distance away from each other. Therefore, both generation goals are achieved.

These generation constraints suggest limiting the search space inside a sphere with radius *r*, and discreting the search space with grids of size *ε*. When *ε*=0.001Å, the accuracy of X-ray crystallography [22] and PDB (protein database) format [23] is reached. Thus, this method is capable of generating all possible idealized structures at the accuracy of X-ray crystallography and PDB format.

Given the limited and discrete search space of each atom, one can generate idealized structure coordinates from the first atom to the last atom. For the first atom, an idealized coordinate lies within a sphere. Thus, the number of generated coordinates is bounded by *O*(1/*ε*^3^). For each generated coordinate $P_{i}^{1}$ of the first atom, an idealized coordinate of the second atom lies on a ball surface with a constant distance to $P_{i}^{1}$. Thus, the number of generated coordinates is bounded by *O*(1/*ε*^2^). For each generated coordinate pair $\left(P_{i}^{1},P_{i}^{2}\right)$ of the first two atoms, an idealized coordinate of the third atom lies on a circle with a constant distances to $P_{i}^{1}$ and $P_{i}^{2}$. Thus, the number of generated coordinates is bounded by *O*(1/*ε*). Similarly, the number of generated coordinates for any of the following atoms is also bounded by *O*(1/*ε*). Moreover, since we round *Ω* dihedral angles to either 0° or 180°, the coordinate of any *C*_*α*_ atom is unique and can be calculated from the coordinates of the previous three atoms.

Therefore, the total number of coordinates generated for all atoms is bounded by *O*(1/*ε*^2*n*+4^) by induction. Here, it is acceptable to assume that *r* is a constant because it is only related to the first atom. For subsequent atoms, we did not limit the search space to be inside the sphere with radius *r* as described above, and thus the actual number of generated coordinates should be much smaller in practice.

### Idealized backbone structure scoring function

Given the generated idealized structures {*P*_*i*_}, we need a scoring function *S*_*BB*_(*P*_*i*_) to find the optimal idealized structure. The scoring function should evaluate not only the similarity between generated idealized structure *P*_*i*_ and target structure *P*_0_, but should also evaluate the free energy of *P*_*i*_, to ensure that *P*_*i*_ is protein-like. Thus, we define our scoring function as follows:

(3) $$\begin{array}{ll} S_{\text{BB}}(P_{i})= & S_{f}(P_{i})-w_{1}D_{\alpha }(P_{i},P_{0})-w_{2}D_{\beta }(P_{i},P_{0})\\ & -w_{3}D_{H}(P_{i},P_{0})-w_{4}D_{\Phi ,\Psi }(P_{i},P_{0}), \end{array}$$

where *w*_*a*_ are the weighting parameters, *S*_*f*_(*P*_*i*_) is the free energy score, *D*_*α*_(*P*_*i*_,*P*_0_) is the root mean square divergence (RMSD) of *C*_*α*_ atoms, *D*_*β*_(*P*_*i*_,*P*_0_) is the RMSD of *C*_*β*_ atoms, *D*_*H*_(*P*_*i*_,*P*_0_) is the RMSD of the hydrogen and oxygen atoms participating in hydrogen bonds, and *D*_*Φ*,*Ψ*_(*P*_*i*_,*P*_0_) is the RMSD of (*Φ*,*Ψ*) dihedral angles.

In our scoring function, the free energy is evaluated by a (*Φ*,*Ψ*) dihedral angle log-odd score as the free energy score *S*_*f*_(*P*_*i*_). Specifically, we discrete the Ramachandran plot into grids of 360 by 360, and draw one plot for each type of amino acid. Then, we calculate the log-odd score $S_{f}(P_{i}^{1,t})$ of idealized structure $P_{i}^{1,t}$ of the first *t* atoms:

(4) $$S_{f}(P_{i}^{1,t})=\underset{5\leq i\leq t,A_{i}=C_{\alpha }}{\sum }\log\frac{P_{AA_{i-3}}(\Phi_{i-3},\Psi_{i-3})}{P_{\text{null}}(\Phi_{i-3},\Psi_{i-3})},$$

where one log-odd score is calculated at each *C*_*α*_ atom (by checking that atom type *A*_*i*_ is *C*_*α*_) for the previous amino acid (represented by the previous *C*_*α*_ atom at *i*−3), $P_{AA_{i-3}}(\Phi_{i-3},\Psi_{i-3})$ is the probability of the grid containing (*Φ*_*i*−3_,*Ψ*_*i*−3_) on the Ramachandran plot of amino acid type *AA*_*i*−3_, and *P*_*null*_(*Φ*_*i*−3_,*Ψ*_*i*−3_) is the probability of the null model with a uniform distribution such that $P_{\text{null}}(\Phi_{i-3},\Psi_{i-3})=\frac{1}{360}\frac{1}{360}$.

Structure similarity is evaluated by other distance matrices in our scoring function. We use *D*_*α*_(*P*_*i*_,*P*_0_) and *D*_*Φ*,*Ψ*_(*P*_*i*_,*P*_0_) to serve as distance metrics to conserve the backbone structures, and *D*_*β*_(*P*_*i*_,*P*_0_) to serve as a distance metric to conserve the side-chain structure compatibilities; we also use *D*_*H*_(*P*_*i*_,*P*_0_) to serve as a distance metric to conserve the hydrogen bonds. Thus, some global dependencies are addressed implicitly by distance matrices $D_{\beta }(P_{i}^{1,t},P_{0}^{1,t})$ and *D*_*H*_(*P*_*i*_,*P*_0_).

### Dynamic programming algorithm

Theoretically, one can calculate scores for all generated idealized structures and find the optimal one with the maximum score. This method works well as long as similar structures always have similar scores. More formally, the method requires the assumption that *D*(*P*_*i*_,*P*_*j*_)≤*ε* ⇒ |*S*_*BB*_(*P*_*i*_)−*S*_*BB*_(*P*_*j*_)|≤*ε*_*s*_, which is reasonable for small *ε*. Note that, since the total number of generated idealized structures is bounded by *O*(1/*ε*^2*n*+4^), this method is computationally expensive. Thus, we introduce a dynamic programming algorithm with a filtering technique to find the optimal idealized structure efficiently.

The dynamic programming algorithm has two assumptions. One assumption is that given two generated idealized structures $P_{i}^{1,t-1}$ and $P_{j}^{1,t-1}$ of the first *t*−1 atoms, such that $D(P_{i}^{t-k,t-1},P_{j}^{t-k,t-1})\leq \varepsilon$, for any generated coordinate $P_{i}^{t}$ of the *t*’th atom, there always exists a generated coordinate $P_{j}^{t}$, such that $D(P_{i}^{t},P_{j}^{t})\leq \varepsilon$. The other assumption is that the scoring function satisfies the additive property, such that $S_{\text{BB}}(P_{i}^{1,t})=S_{\text{BB}}(P_{i}^{1,t-k})⊕S_{\text{BB}}(P_{i}^{t-k+1,t})$, under some addition operator ⊕.

We observed that counter examples of the first assumption when *k*≥5 are rare, though counter examples do exist theoretically. The second assumption holds for our scoring function. Distance matrices $D_{\alpha }(P_{i}^{1,t},P_{0}^{1,t})$, $D_{\beta }(P_{i}^{1,t},P_{0}^{1,t})$, $D_{H}(P_{i}^{1,t},P_{0}^{1,t})$ and $D_{\Phi ,\Psi }(P_{i}^{1,t},P_{0}^{1,t})$ satisfy the additive property because RMSD $D_{\text{RMS}}(P_{i}^{1,t},P_{0}^{1,t})$ satisfies the additive property:

(5) $$\begin{array}{l} \;D_{\text{RMS}}(P_{i}^{1,t},P_{0}^{1,t})\\ =D_{\text{RMS}}(P_{i}^{1,t-k},P_{0}^{1,t-k})⊕D_{\text{RMS}}(P_{i}^{t-k+1,t},P_{0}^{t-k+1,t})\\ =\sqrt{\frac{D_{\text{RMS}}^{2}(P_{i}^{1,t-k},P_{0}^{1,t-k})(t-k)+D_{\text{RMS}}^{2}(P_{i}^{t-k+1,t},P_{0}^{t-k+1,t})k}{t}}. \end{array}$$

Moreover, the free energy score $S_{f}(P_{i}^{1,t})$ satisfies the additive property as follows:

(6) $$\begin{array}{ll} S_{f}(P_{i}^{1,t}) & =S_{f}(P_{i}^{1,t-k})⊕S_{f}(P_{i}^{t-k+1,t})\\ & =S_{f}(P_{i}^{1,t-k})+S_{f}(P_{i}^{t-k+1,t}). \end{array}$$

The second assumption is fundamental to our dynamic programming algorithm. By induction, the first assumption implies that if $D(P_{i}^{t-k,t-1},P_{j}^{t-k,t-1})\leq \varepsilon$, for any generated idealized structure $P_{i}^{t,n}$, there always exists a generated idealized structure $P_{j}^{t,n}$ such that $D(P_{i}^{t,n},P_{j}^{t,n})\leq \varepsilon$. Recall that the scoring function assumes that $D(P_{i}^{t,n},P_{j}^{t,n})\leq \varepsilon \;\Rightarrow \;|S_{\text{BB}}(P_{i}^{t,n})-S_{\text{BB}}(P_{j}^{t,n})|\leq \varepsilon_{s}$, and thus there is $S_{\text{BB}}(P_{i}^{t,n})\approx S_{\text{BB}}(P_{j}^{t,n})$. If $S_{\text{BB}}(P_{i}^{1,t-1})\geq S_{\text{BB}}(P_{j}^{1,t-1})$, there is approximately $S_{\text{BB}}(P_{i})=S_{\text{BB}}(P_{i}^{1,t-1})⊕S_{\text{BB}}(P_{i}^{t,n})\geq S_{\text{BB}}(P_{j}^{1,t-1})⊕S_{\text{BB}}(P_{j}^{t,n})=S_{\text{BB}}(P_{j})$. Therefore, if $D(P_{i}^{t-k,t-1},P_{j}^{t-k,t-1})\leq \varepsilon$ and $S_{\text{BB}}(P_{i}^{1,t-1})\geq S_{\text{BB}}(P_{j}^{1,t-1})$, there is no need to generate $P_{j}^{t,n}$ to find an approximately optimal solution.

Based on this observation, we developed a novel dynamic programming algorithm. Idealized structures are still generated as previously described, but the generation process is stopped for some idealized structures if we know it cannot lead us to the optimal one. First, the search space for each atom of the target protein is discretized to grids of size *ε*. When generating coordinates for atom *t*, if $P_{i}^{t-k+1,t}$ and $P_{j}^{t-k+1,t}$ are located in the same grid set $G_{g}^{t-k+1,t}$, we know that there is no need to continue the generation process on the lower scoring one of $P_{i}^{1,t}$ and $P_{j}^{1,t}$. Thus, we define the dynamic programming table $T_{\text{BB}}(t,G_{g}^{t-k+1,t})$ to be the optimal idealized structure for each observed tail grid set $G_{g}^{t-k+1,t}$ as follows:

(7) $$\begin{array}{l} \left\{\begin{array}{l} T_{\text{BB}}(t,G_{g}^{t-k+1,t})=\underset{i,j}{\max}T_{\text{BB}}(t-1,G_{i}^{t-k,t-1})\\ \;⊕S_{\text{BB}}(P_{j}^{t})\\ T_{\text{BB}}(k,G_{g}^{1,k})=\underset{i}{\max}S_{\text{BB}}(P_{i}^{1,k}) \end{array}\right), \end{array}$$

where $G_{g}^{t-k+1,t-1}=G_{i}^{t-k+1,t-1}$, $P_{j}^{t-k+1,t}\in G_{g}^{t-k+1,t}$ and $S_{\text{BB}}(P_{j}^{1,t-1})⊕S_{\text{BB}}(P_{j}^{t})=S_{\text{BB}}(P_{j}^{1,t})$. Thus, the dynamic programming table can be calculated from the first atom to the last atom. Finally, the optimal idealized structure is the one with the highest score $\underset{g}{\max}G_{g}^{3n-k+1,3n}$.

The run-time complexity of our dynamic programming algorithm depends on the value of *k*. To keep all possible (*Φ*,*Ψ*) dihedral angles of the previous residue when generating *C*_*α*_ atoms, we have to choose *k*≥5. For speed, we choose *k*=5 in our implementation. In this case, the number of score calculations required to calculate $T_{\text{BB}}(t,G_{g}^{t-4,t})$ is no more than the maximum number of coordinates sampled for six consecutive backbone atoms. Recall that there are exactly two *C*_*α*_ atoms in six consecutive backbone atoms, and the *Ω* dihedral angle is rounded. Thus, the coordinate of one *C*_*α*_ atom can be calculated from the coordinates of the other *C*_*α*_ atom and the two atoms between them. For this reason, the maximum number of sampled coordinates is bounded by *O*(1/*ε*^8^). Moreover, the number of score calculations required to calculate $T_{\text{BB}}(k,G_{g}^{1,k})$ is no more than the maximum number of possible coordinates sampled for five consecutive backbone atoms, which is also *O*(1/*ε*^8^). Therefore, the run-time complexity of our dynamic programming algorithm is *O*(*n*/*ε*^8^).

To increase the speed for the dynamic programming algorithm, we applied an additional filtering technique to remember only the highly scored idealized structures. Specifically, the algorithm only remembers the optimal idealized structure for the top *m* scored tail configurations instead of all possible conformations. Thus, the run-time complexity is reduced to *O*(*nm*/*ε*). This approach works well in practice because an optimal idealized structure with a long poorly scored fragment is rare. Thus, we assumed that the local quality of the idealized structure should be reasonably high (in the top *m* score list).

## Protein side-chain structure idealization

After the backbone structure of the target protein has been idealized, we begin to idealize the side-chain structures. When doing this, the idealized backbone structure is considered to be rigid. This approach is widely accepted because previous research suggests that the backbone conformation is archived before the side-chain conformations are archived [14]. After the side-chain idealization, we should have a complete idealized protein structure with all of the backbone and the side-chain structures idealized.

Protein side-chains suffer from low quality problems when determining protein structures. This is mainly because side-chains are not as stable as backbones, and they are more likely to have disorder problems than are backbones in crystals [22]. Thus, the target side-chain structure might be a poor reference for defining the search space and for evaluating the structure similarity score for generated idealized side-chain structures. To address this, we perform an exhaustive search on the entire feasible torsion angle space, instead of the limited torsion angle space, around the target side-chain structure.

Our side-chain idealization method assumes that the side-chain conformations of different residues are independent of each other. Otherwise, all residues with dependencies have to be generated together and the run-time complexity increases exponentially to the number of atoms involved. Moreover, the *N*_*η*1_−*C*_*ζ*_−*N*_*ε*_−*C*_*δ*_ and the *N*_*η*2_−*C*_*ζ*_−*N*_*ε*_−*C*_*δ*_ torsion angles of arginine residues are rounded to be either 0° or 180°. Then, the degree of freedom of the search space for each residue is at most four and it is now practical to perform an exhaustive search for each residue independently.

To find the optimal idealized side-chain structure, we design a new scoring function involving the similarity among the generated idealized side-chain structures and the target side-chain structures, and the free energy of the generated idealized side-chain structures. Let *P*_0_ be the target side-chain structure of some residue, and *P*_*i*_ for all *i*>0 be a generated idealized side-chain structure of the same residue. Then, the scoring function *S*_*SC*_(*P*_*i*_) is defined:

$$S_{\text{SC}}(P_{i})=S_{f}(P_{i})-w_{1}D_{H^{\prime }}(P_{i},P_{0})-w_{2}D_{\chi }(P_{i},P_{0}),$$

where *w*_*k*_ are the weighting parameters, *S*_*f*_(*P*_*i*_) is the free energy score, $D_{H^{\prime }}(P_{i},P_{0})$ is the root mean square divergence (RMSD) of all non-hydrogen atoms, and *D*_*χ*_(*P*_*i*_,*P*_0_) is the RMSD of *χ* torsion angles.

In our scoring function, the free energy score *S*_*f*_(*P*_*i*_) is defined as a simple *χ* torsion angle log-odd score, which is similar to the free energy score of our backbone scoring function. Moreover, the log-odd score is based on the popular backbone dependent rotamer library downloaded from Dunbrack’s lab [24]. Certainly, other local free energy scores can be adopted here. Similar to the backbone scoring function, $D_{H^{\prime }}(P_{i},P_{0})$ and *D*_*χ*_(*P*_*i*_,*P*_0_) serve as distance metrics to conserve the side-chain structure.

## Result

To study the protein structure idealization problem and its applications, we implemente our protein structure idealization algorithm. In our implementation, we use the mean bond lengths and angles that had been reported in [2] as the ideal bond lengths and angles, respectively. When idealizing the protein backbone structure, we set the search space radius of an atom as *r*=1.6Å and the discrete grid size as *ε*=*r*/5. We find that *m*=50,000 had a reasonable balance between speed and accuracy. When idealizing the protein side-chain structure, we set the search space of a rotamer dihedral angle to be within 3*σ* distance from the mean value, where *σ* is the STDEV of the rotamer dihedral angle, and we set the discrete grid size to be 10°. We also refine the idealized structure by iteratively reducing the search space and the discrete grid size by a constant factor of 0.5. Since finding the best scoring function for the protein structure idealization is out of the scope of this paper, we set all weights *w*_*a*_=1.0 for all *a* in our scoring function.

### PDB protein structure idealization

In this experiment, we addressed how accurately it is possible to model protein structures with dihedral angles. We idealized high resolution protein structures with low sequence identities of the CULLPDB_PC30_RES1.6_R0.25 data set [18,19]. In fact, the CULLPDB_PC30_RES1.6_R0.25 data set is the complete set of X-ray protein structures in PDB [17] with a sequence identity cutoff of 30*%*, a resolution cutoff of 1.6Å, and an *r* factor cutoff of 0.25. In summary, the data set contains 1898 proteins with an average length of 227 residues, as downloaded on June 6, 2008.

**Figure 1:**
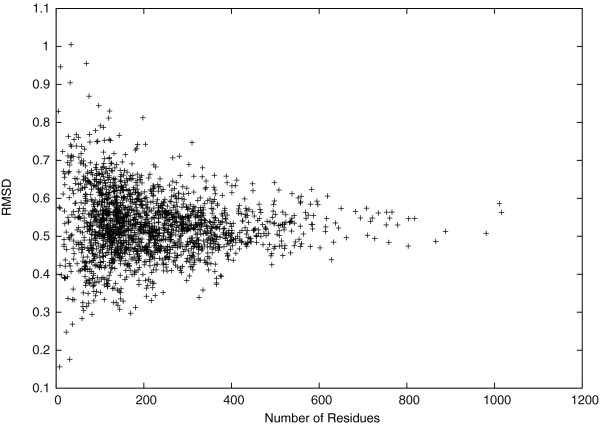
***C***_***α***_**-RMSD.**

To show that the idealized and the target backbone structures are very similar, we calculated the *C*_*α*_-RMSD as shown in Figure 1. The *C*_*α*_-RMSD is a popular distance metric to evaluate the backbone distance between two protein backbone structures. The result shows that most distances between the idealized and the target backbone structures are small with mean 0.53Å and STDEV 0.08Å. Specifically, the smallest *C*_*α*_-RMSD reaches 0.16Å, and 90*%* of the *C*_*α*_-RMSDs are smaller than 0.63Å. Moreover, the *C*_*α*_-RMSD is upper bounded by 1.00Å, although the search space radius for each atom is set to be 1.6Å. This result is consistent with the result of checking (*Φ*,*Ψ*) dihedral angles, where the average difference between the idealized and the target (*Φ*,*Ψ*) dihedral angles is as small as 0.08°. Therefore, it is possible to model protein backbone structures in CULLPDB_PC30_RES1.6_R0.25 accurately using only *Φ* and *Ψ* dihedral angles.

**Figure 2:**
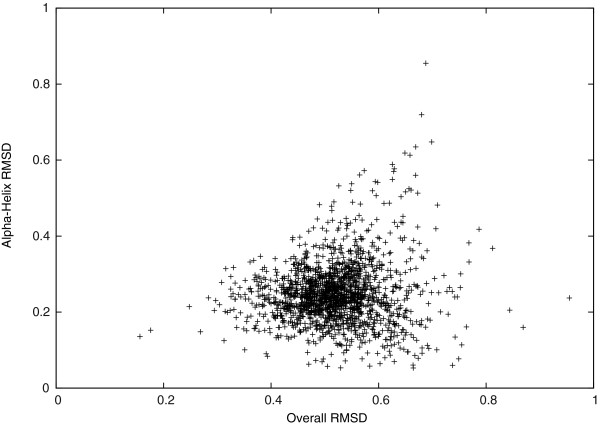
***C***_***α***_**-RMSD of all regions v.s. *α*-helix regions.**

**Figure 3:**
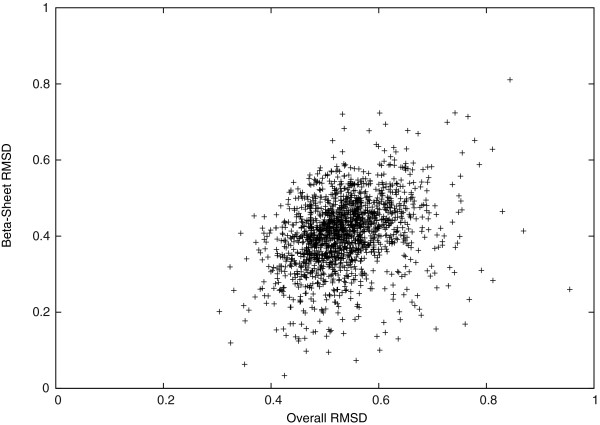
***C***_***α***_**-RMSD of all regions v.s. *β*-sheet regions.**

**Figure 4:**
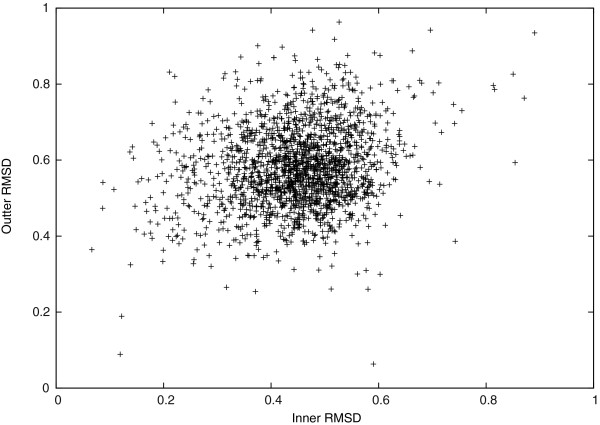
***C***_***α***_**-RMSD of inner regions v.s. outer regions.**

We studied the *C*_*α*_-RMSD further in different regions of the target protein structures. In Figures 2 and 3, we see that the *C*_*α*_-RMSD of the *α*-helix and the *β*-sheet regions are smaller than that of the complete protein by 0.28Å and 0.12Å, respectively. Indeed, these regions are more restricted because of using *D*_*H*_(*P*_*i*_,*P*_0_) to conserve hydrogen bonds of *α*-helices and *β*-sheets in our scoring function. We also observe that the *C*_*α*_-RMSD of residues that are closer to the geometric center of a target protein structure is 0.13Å smaller on average than the *C*_*α*_-RMSD of the other residues that are farther, as shown in Figure 4. Thus, the inner residues tend to be closer to the idealization state than are the outer residues. We did not observe any significant differences on the *C*_*α*_-RMSD between the buried and the exposed regions.

**Figure 5:**
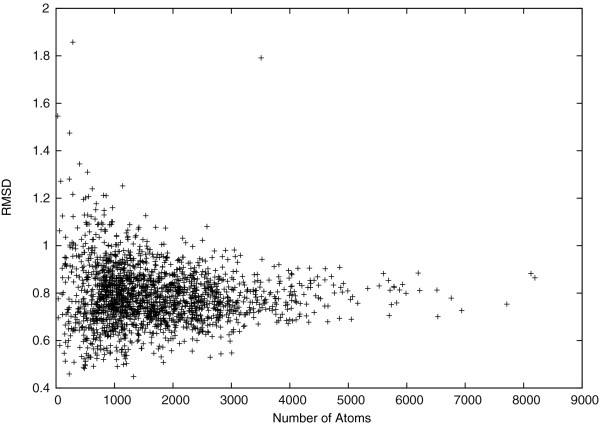
All-atom RMSD.

We also calculated the all-atom RMSD to show that the idealized and the target structures are very similar. In Figure 5, we see that most distances between the idealized and the target structures are small, with mean 0.79Å and STDEV 0.13Å. Moreover, the smallest all-atom RMSD reaches 0.45Å, and 90*%* of the all-atom RMSDs are smaller than 0.94Å. Note that both the *C*_*α*_-RMSD and the all-atom RMSD between the idealized and the target structures tend to be stable when the target protein is long. Therefore, it is possible to model protein structures accurately with only *Φ*, *Ψ*, and *χ* dihedral angles.

**Figure 6:**
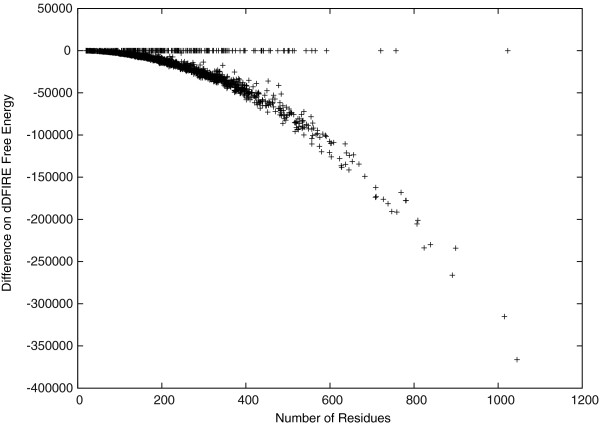
Protein backbone free energy (calculated by dDFIRE).

The idealized backbone structures are also favored in terms of free energy. This is shown by checking the free energy differences between the idealized and the target protein backbone structures in Figure 6. Here, we calculate the free energy using dDFIRE [20,21], and observe that the dDFIRE free energy of most idealized backbone structures are significantly better than are those of the target backbone structures. For the rest without significant improvements, the difference is close to zero. This may be the result of some tight thereochemical restraints used in existing X-ray structure refinement programs [15,16]. It is also interesting that the observed free energy improvements are clearly not independent from the protein length. The figure suggests that the free energy difference has a square dependence on the protein length.

**Figure 7:**
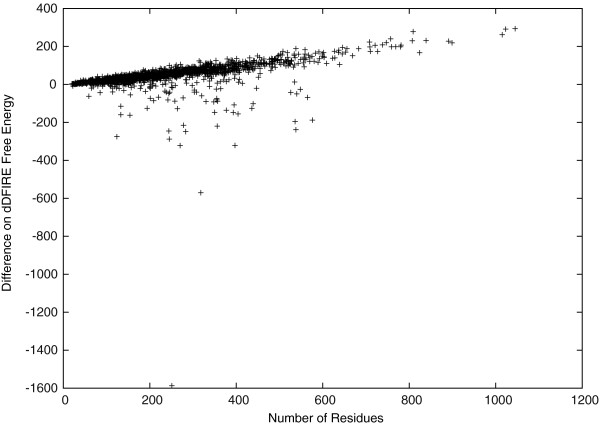
Protein all-atom free energy (calculated by dDFIRE).

After idealizing the side-chain structures, the free energy is either improved by a relatively biger amount or worsened by a relatively smaller amount as shown in Figure 7. Unfortunately, in most cases, the free energy is worsened slightly but is still in a stable state with negative values. Again, here we used dDFIRE [20,21] to calculate the free energy. We observed that the dDFIRE free energy is improved for 90 or 4.74*%* of the idealized protein structures and is worsened slightly by 44 on average. Moreover, the dDFIRE free energy is improved by 1585 in the best case, and worsened by 293 in the worst case. The figure also suggests that the free energy difference has a linear dependence on the protein length.

Several side-chain prediction tools have been proven to predict accurate side-chain structures from native backbone structures [8,9,25,26]. However, these tools does not perform well when predicting side-chain structures from predicted backbone structures. To address this, we compared the predicted side-chain structures given the native backbone structures and those given the predicted backbone structures in terms of free energy. Here, we treat the idealized backbone structures of the CULLPDB_PC30_RES1.6_R0.25 data set as those which are best possibly predicted. Moreover, we used SCWRL4 [9] to predict side-chain structures and dDFIRE [20,21] to calculate free energies. The result shows that the free energy is worsened slightly by 43 if the predicted backbone structures are used. We do not think this difference is significant to side-chain prediction. Certainly, more experiments will show if this is conclusive.

Finally, we study the effects of idealization on hydrogen bonds. As shown in Table 1, we compare the number of hydrogen bonds detected by the DSSP program [27,28]. Here, only differences of the most popular types of hydrogen bonds are included. We note that the total number of hydrogen bonds is increased by 1.59*%* or 0.012 per residue after idealization. Specifically, the effects of idealization on hydrogen bonds of *β* bridges is minor, and the loss of the hydrogen bonds on *α* helices is reasonably controlled under 1.48*%*. Interestingly, the idealized backbone structures have significantly more 2_7_ ribbons. The reason behind this observation remains open.

**Table 1:** DSSP hydrogen bond differences before and after idealization

**Type**	**Count difference**	**Percent difference**
Parallel Bridge	9	0.04%
Antiparallel Bridge	-211	-0.37%
2_7_ Helix	7080	26.46%
3_10_ Helix	-1018	-2.35%
*α* Helix	-1644	-1.48%
*Π* Helix	-82	-1.27%
All	5183	1.85%

In summary, we demonstrate that using dihedral angles with ideal bond lengths and angles is capable of modeling protein structures that are highly similar to the ones in CULLPDB_PC30_RES1.6_R0.25 [18,19]. Since CULLPDB_PC30_RES1.6_R0.25 is the complete set of PDB protein structures satisfying the high resolution and the low sequence identity constraints, it is reasonable to extend the conclusion to all protein backbone structures. A positive side effect is that idealization improves backbone free energy, while most hydrogen bonds are conserved.

### NMR protein structure refinement

In this experiment, we demonstrate an application of the protein structure idealization problem in NMR by idealizing 32 NMR protein structures. The NMR protein structures were randomly chosen from PDB [17] with a sequence identity cutoff of 30*%* and a gapless fragment length cutoff of 80 residues. In cases of multiple chains or models of some NMR protein structures, only the first chain from the first model is used in this experiment. This addition to the conclusion of the previous experiment shows that poor (*Φ*,*Ψ*) dihedral angles of the NMR protein structures are improved by idealizing them.

To demonstrate this, we compared the percentage of favored (*Φ*,*Ψ*) dihedral angles calculated by PROCHECK [29] in Table 2. After idealization, we see that 19 out of 32 NMR protein structures have more favored (*Φ*,*Ψ*) dihedral angles. Overall, the percentage is increased by 4.34*%* on average and 27.30*%* in the best case, which is closer to the minimum percentage of 90*%* expected in a good quality model [29].

**Table 2:** **The percentages of the favored *(Φ,Ψ) *dihedral angles of *32 *NMR protein structures before and after idealization**

**PDB**	**Native**	**Ideal**	**Diff**	**PDB**	**Native**	**Ideal**	**Diff**
1SSK	44.6%	71.9%	27.3%	2LBN	59.7%	77.6%	17.9%
2KQP	62.9%	80.0%	17.1%	1WPI	64.4%	81.4%	17.0%
1EXE	60.5%	76.7%	16.2%	2LNV	58.6%	72.4%	13.8%
1X6F	64.1%	73.1%	9.0%	2L6B	72.2%	81.1%	8.9%
2GFU	72.3%	80.4%	8.1%	1PC2	79.3%	87.4%	8.1%
2LMR	79.7%	87.0%	7.3%	2KA0	72.6%	78.3%	5.7%
2L3O	71.3%	76.9%	5.6%	1O1W	67.2%	72.1%	4.9%
2CQ9	78.3%	82.6%	4.3%	2RQA	72.0%	75.4%	3.4%
2D86	89.0%	92.1%	3.1%	1NTC	80.5%	83.1%	2.6%
2JZT	76.6%	79.0%	2.4%	2CZN	76.5%	76.5%	0.0%
1RCH	75.4%	74.6%	-0.8%	2JU1	77.1%	75.9%	-1.2%
2KV7	85.5%	84.2%	-1.3%	2JT2	83.6%	81.5%	-2.1%
2KYW	83.8%	81.1%	-2.7%	2OSR	82.7%	80.0%	-2.7%
2L6M	81.7%	78.5%	-3.2%	2CU1	81.1%	77.8%	-3.3%
1AJ3	93.3%	88.8%	-4.5%	1WI5	84.0%	78.0%	-6.0%
1NMW	85.0%	78.0%	-7.0%	2LBV	83.9%	74.8%	-9.1%

Note that for those NMR protein structures that already have more than approximately 75*%* of favored (*Φ*,*Ψ*) dihedral angles, idealization harms the percentage by −0.85*%* on average. There are at least two reasons for this. First, our free energy score *S*_*f*_(*P*_*i*_) is calculated from a data set that is different from the one used by PROCHECK. In fact, we used 1898 protein structures of the CULLPDB_PC30_RES1.6_R0.25 data set [18,19], while PROCHECK used 118 protein structures, with a resolution cutoff of 2.0Å and an *r* factor cutoff of 0.20 [29]. Although the percentages of favored (*Φ*,*Ψ*) dihedral angles are decreased in Table 2, our free energy scores of proteins 1WI5, 1NMW, and 2LBV are increased by 0.22, 1.35, and 0.31, respectively, after idealization. Second, our implementation is trying to optimize our scoring function *S*_*BB*_(*P*_*i*_), instead of optimizing only the free energy score. Thus, it is possible to see decreased free energy scores after idealization, especially when the target protein structure has a high percentage of favored (*Φ*,*Ψ*) dihedral angles.

**Figure 8:**
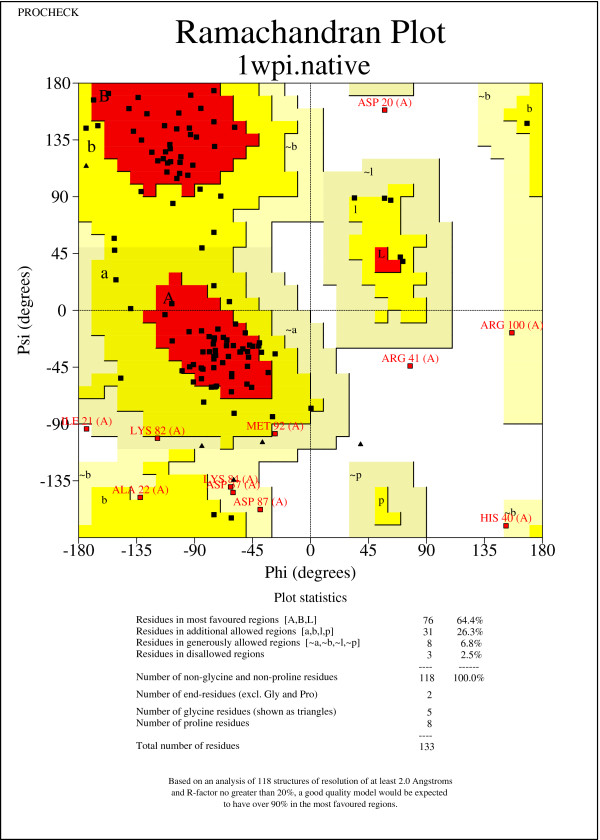
Ramachandran plot of the native NMR protein structure 1WPI.

**Figure 9:**
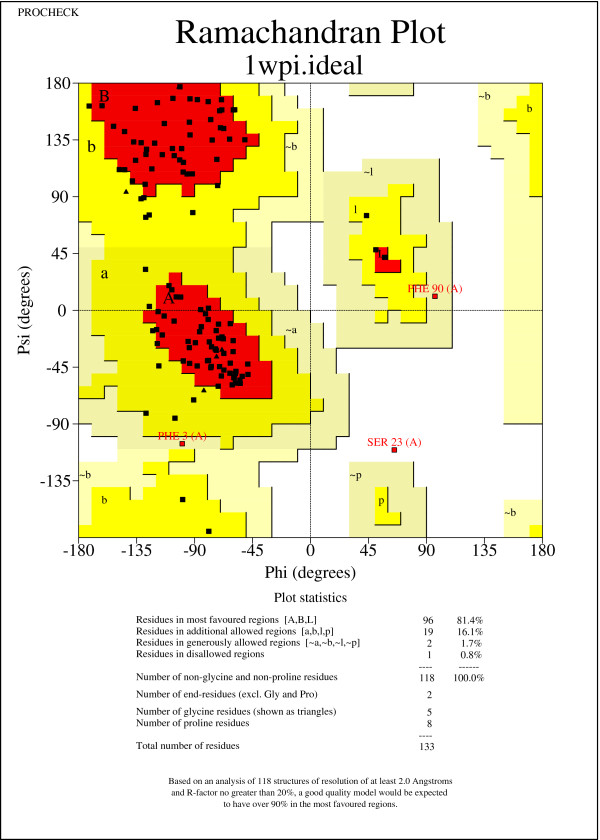
Ramachandran plot of the idealized NMR protein structure 1WPI.

Our conclusion is further supported by the case study of the NMR structure with PDB ID 1WPI [30]. From the Ramachandran plots drawn by PROCHECK [29] in Figures 8 and 9, we find that (*Φ*,*Ψ*) dihedral angles tend to move towards favored regions. Specifically, the native structure contains only 64.4*%* of (*Φ*,*Ψ*) dihedral angles in favored regions, while the idealized structure contains a significantly improved percentage of 81.4*%* of (*Φ*,*Ψ*) dihedral angles in favored regions. Moreover, the native structure contains three (*Φ*,*Ψ*) dihedral angles that are not in any feasible areas of the Ramachandran plot. However, there is only one such case found in the idealized structure. Thus, two infeasible (*Φ*,*Ψ*) dihedral angles are fixed by the (*Φ*,*Ψ*) dihedral angle log-odd score. Here, we did not, but certainly can, implement a hard constraint to disallow any infeasible (*Φ*,*Ψ*) dihedral angles.

In summary, we have demonstrated that protein structure idealization can be used to improve poor (*Φ*,*Ψ*) dihedral angles of protein pseudo-structures. These protein pseudo-structures can either be predicted or be experimentally determined. More applications of the protein structure idealization problem will be studied.

## Conclusion

We have introduced the protein structure idealization problem and performed our first attempt to solve it. The experiment results show that idealized structures always exist with small changes on the coordinates. Furthermore, the idealized backbone structures have significantly better free energy and (*Φ*,*Ψ*) dihedral angle distributions. Therefore, protein structures can be modeled accurately with dihedral angles and ideal bond lengths and angles, and it is feasible to predict protein backbone and side-chain structures by searching the dihedral angle space.

Our protein structure idealization algorithm may be improved in several ways. Since our scoring functions are very simple with all weights *w*_*a*_=1.0 in the current implementation, there is space for improvements. We are also looking forward to adding protein-ligand interaction energy to our scoring function and to studying the effect of idealization on protein-ligand interactions. Moreover, since some atoms are more flexible than others, we can also set different search spaces for different atoms in our algorithm. For example, when idealizing X-ray protein structures, the search space of each atom could be selected according to its B-factor. We can also adopt a divide-and-conquer algorithm in our algorithm to find the global, rather than local, optimal idealized structure. Specifically, we can divide the protein structure into small fragments, idealize each fragment separately, and merge idealized fragments. The key is to divide the protein structure by a tree decomposition of the interaction graph and to remember the optimal idealized fragment for each possible configuration of atoms with interactions to external atoms. Similar ideas have already been used successfully to improve the speed and the accuracy of backbone and side-chain structure predictions [8,9,25,26,31,32].

Our protein structure idealization algorithm can also correct modelling errors of protein structures in PDB [17]. In fact, previous research indicates that many bond conformations and side-chain rotamers are likely incorrect in PDB, and it is useful to have an automated mechanism to fix these problems [33,34]. Thus, we can address these problems by idealizing all protein structures in PDB with our protein structure idealization algorithm and using our specially tuned scoring functions.

The idealized version of the PDB [17] provides new protein structure references to study protein structures and functions. For example, we can rebuild fragment and rotamer libraries based on the idealized PDB. It would then be more intuitive to use the idealized fragment or rotamer libraries in the protein backbone or side-chain structure prediction algorithms searching the dihedral angle space. Thus, we expect to see some improvements of the accuracy of these algorithms with the idealized fragment and rotamer libraries. Therefore, we also provide a new approach for discovering unusual atoms and bonds by comparing the idealized and the original PDB structures. Although most of these unusual atoms and bonds are due to errors, we expect to discover some biochemical insights that assist in understanding protein functions.

## Competing interests

The authors declare that they have no competing interests.

## Authors’ contributions

The algorithm implementation and all experiments were done by XC; the protein structure idealization algorithm was designed by XC and SCL; the CULLPDB_PC30_RES1.6_R0.25 experiment was designed by XC, SCL and DB; the NMR experiment was designed by XC and BA; the project was directed by ML; All authors read and approved the final manuscript.
